# Artificial Intelligence and Computational Issues in Engineering Applications

**DOI:** 10.3390/e25010005

**Published:** 2022-12-21

**Authors:** Karolina Grabowska, Jaroslaw Krzywanski, Marcin Sosnowski, Dorian Skrobek

**Affiliations:** Faculty of Science and Technology, Jan Dlugosz University in Czestochowa, Armii Krajowej 13/15, 42-200 Czestochowa, Poland

High-performance supercomputers and emerging computing clusters created in research and development centres are rapidly increasing available computing power, which scientists are eager to use to implement increasingly advanced computing methods [1]. Thus, computationally demanding artificial intelligence algorithms and computational fluid dynamics methods are used more widely to consider complex engineering issues and verify and provide new information on entropy or information theory concepts [2,3]. Therefore, it is crucial to conduct time-consuming optimization analyses, as entropy generation accomplished by exergy destruction is a characteristic of complex systems. Their irreversibilities should be reduced to increase their performance. Optimization via modelling and simulations allows an increase in a system’s efficiency.

The Special Issue titled “Artificial Intelligence and Computational Issues in Engineering Applications” aimed to bring together research related to the modelling of complex systems in a wide variety of engineering applications. The summary of the contributions accepted for the Special Issue of *Entropy* belonging to the section “Multidisciplinary Applications” is presented in this paper in order of the publication date.

Sylvanus Gbenou et al. [4] submitted a very interesting review concerning the recent status of thermochemical heat storage (TCHS) processes and applications. The authors reviewed the latest contributions to thermochemical heat storage (TCHS) technology and revealed four main branches that could significantly contribute to the field. These were the control of the processes to store or release heat, a perfect understanding and designing of the materials used for each storage process, the good sizing of the reactor, and the mastery of the whole system connected to design an efficient system. The paper emphasized that the above-mentioned fields constitute a very complex area of investigation, and most of the works focus on one of the branches to deepen their research. Moreover, the authors noticed that the analysed technology is still not mature, and, up to now, no definitive, efficient, autonomous, practical, and commercial TCHS device is available. Several issues that impede the maturity of the technology have been clearly highlighted. These are the limited number of research works dedicated to the topic, the simulation results that are too illusory and impossible to implement in real prototypes, and the endless problem of heat and mass transfer limitation. Moreover, papers presenting incomplete systems analysis, i.e., simulation works without experiments or experiments without prior simulation study, were identified as a severe problem. To summarize, the above paper provides insights and recommendations to better analyse and solve the problems that still challenge TCHS technology.

An automatic extraction algorithm for crop images based on Mask RCNN has been proposed by Wang et al. [5]. The authors presented a computational algorithm based on deep learning. The first step was setting the Fruits 360 Dataset label with Labelme. Then, the Fruits 360 Dataset was preprocessed. Next, the data were divided into a training set and a test set. Additionally, an improved Mask RCNN network model structure was established using the PyTorch 1.8.1 deep learning framework, and path aggregation and features are added to the network design enhanced functions, optimized region extraction network, and feature pyramid network. The spatial information of the feature map was saved by the bilinear interpolation method in ROIAlign. Finally, the edge accuracy of the segmentation mask was further improved by adding a micro-fully connected layer to the mask branch of the ROI output, employing the Sobel operator to predict the target edge, and adding the edge loss to the loss function. The achieved experimental results demonstrate that the improved Mask RCNN algorithm proposed in this paper is better in the precision, recall, average precision, mean average precision, and F1 scores of crop image extraction results than other conventional image extraction algorithms described in this study.

Fawad Khan et al. [6] examined a unipolar electrohydrodynamic (UP-EHD) pump flow with known electric potential at the emitter and zero electric potential at the collector. The developed model was designed for electric potential, charge density, and electric field. The dimensionless parameters, namely the electrical source number (Es), the electrical Reynolds number (ReE), and the electrical slip number (Esl), were considered with wide ranges of variation to analyse the UP-EHD pump flow. To interpret the pump flow of the UP-EHD model, a new design numerical solver is proposed for the solutions of fluid mechanics problem, unipolar EHD model, with the help of hybridization of supervised and unsupervised methods, i.e., global search technique sine–cosine algorithm (SCA) and local search technique sequential quadratic programming under the influence of an artificial neural network. The developed method is abbreviated as ANN-SCA-SQP in the paper. The authors have demonstrated the superiority of the technique by comparing the achieved solution with reference solutions. The technique was executed for one hundred independent experiments for a large data set. The efficiency has been evaluated through performance operators and convergence plots.

In the framework of the next submitted contribution, Ahmad Khan et al. [7] analysed the mathematical model of various nonlinear oscillators arising in different fields of engineering. In this study, the feedforward neural networks (NNs) based on the backpropagated Levenberg–Marquardt algorithm (BLMA) were used to investigate the approximate solutions for different variations in oscillators. The authors generated a data set for different problem scenarios for the supervised learning of BLMA by the Runge-Kutta method of order 4 (RK-4) with the “NDSolve” package in Mathematica. The value of the approximate solution by the NN-BLMA method was attained by employing the processing of testing, training, and validation of the reference data set. For each model, convergence analysis, error histograms, regression analysis, and curve fitting have been considered to study the robustness and accuracy of the design scheme.

Thermodynamics irreversibilities studies of oxy-fuel diffusion flames are presented by Yan et al. [8]. The impact of the oxygen concentration in the oxidizer on the entropy generation was analysed. The authors of the work demonstrated that thermodynamic analysis can be used to evaluate the irreversibility of the combustion process and improve energy utilization efficiency. The work contains an advanced simulation of the combustion process of a laminar oxy-fuel diffusion flame, and the entropy generation due to the irreversibilities of the radiation, the heat conduction, the heat convection, the mass diffusion, and the chemical reaction processes were calculated. The results presented in this study indicated that, as the oxygen concentration in the oxidizer increases, the radiative entropy generation first increases and then decreases, and the mass diffusion entropy generation, the convective and conductive entropy generation, the chemical entropy generation, and the total entropy generation gradually increase.

Luo et al. [9] proposed a novel edge server placement algorithm based on deep q-network and reinforcement learning, dubbed DQN-ESPA, which can achieve optimal placements without relying on previous placement experience. According to the authors’ considerations, the necessity of developing a new approach results from the imperfections of conventional methods, which have profound defect implications such as poor scalability, local optimal solutions, and parameter tuning difficulties. In the authors’ proposed DQN-ESPA method, the edge server placement problem is modelled as a Markov decision process, which is formalized with the state space, action space, and reward function, and it is subsequently solved using a reinforcement learning algorithm. The experimental results presented in the paper and achieved using real datasets from Shanghai Telecom indicate that DQN-ESPA outperforms state-of-the-art algorithms such as the simulated annealing placement algorithm, Top-K placement algorithm, K-Means placement algorithm, and random placement algorithm. After comprehensive consideration of access delay and workload balance, results proved that DQN-ESPA implementation allowed for achieving up to 13.40% and 15.54% better placement performance for 100 and 300 edge servers, respectively.

Another paper published in the Special Issue dealt with the application of mobile robots in space exploration. Luo et al. [10] simulated the space exploration issue of mobile robots based on the decision-making process of the cognitive architecture of Soar, and three space exploration heuristic algorithms (HAs) were further proposed by the authors based on the model to improve the robot’s exploration speed. The experimental tests were conducted based on the Easter environment, and the results showed that HAs had improved the exploration speed of the Easter robot at least 2.04 times that of the original algorithm in Easter. In this way, the effectiveness of the proposed robot space exploration strategy and the corresponding heuristic algorithms have been verified. Thus, the presented contribution of the paper still stays in line with the raised at the start conclusion that it is crucial to deal effectively with time-consuming optimization analyses. It also seems that imputing expert knowledge in the model can effectively speed up the calculation time without compromising the model’s accuracy [11,12,13].

As can be seen above, the original research articles, as well as review articles focused on optimization by artificial intelligence (AI) algorithms on computational and entropy issues, have been submitted to the Special Issue. The authors of the published papers are representatives of prestigious research universities from around the world, such as China, Chile, Cameroon, Pakistan, Saudi Arabia, and Colombia.

The thematic diversity of the submitted works and their interdisciplinary character indicate that artificial intelligence and computational issues are implemented in various engineering applications in a wide range of industries and research areas. Therefore, creating such universal Special Issues helps to recognize and present the versatility of advanced computing methods.
